# HIV pre-exposure prophylaxis and health and community systems in the Global South: Thailand case study

**DOI:** 10.7448/IAS.18.4.19953

**Published:** 2015-07-20

**Authors:** Donn Colby, Kriengkrai Srithanaviboonchai, Suphak Vanichseni, Sumet Ongwandee, Nittaya Phanuphak, Michael Martin, Kachit Choopanya, Suwat Chariyalertsak, Frits van Griensven

**Affiliations:** 1 SEARCH, Thai Red Cross AIDS Research Centre, Bangkok, Thailand; 2 Thai Red Cross AIDS Research Centre, Bangkok, Thailand; 3 Research Institute for Health Sciences, Chiang Mai University, Chiang Mai, Thailand; 4 Department of Community Medicine, Faculty of Medicine, Chiang Mai University, Chiang Mai, Thailand; 5 Bangkok Tenofovir Study Group, Bangkok, Thailand; 6 Bureau of AIDS, TB and STI, Department of Disease Control, Ministry of Public Health, Nonthaburi, Thailand; 7 Thailand Ministry of Public Health – U.S. Centers for Disease Control and Prevention Collaboration, Nonthaburi, Thailand; 8 U.S. Centers for Disease Control and Prevention, Division of HIV/AIDS Prevention, Atlanta, GA, USA; 9 Division of Preventive Medicine and Public Health, University of California – San Francisco San Francisco, CA, USA

**Keywords:** HIV, PrEP, Thailand, prevention, prophylaxis

## Abstract

**Introduction:**

Pre-exposure prophylaxis (PrEP) is recommended by the World Health Organization as an effective method of HIV prevention for individuals at risk for infection. In this paper, we describe the unique role that Thailand has played in the global effort to combat the HIV epidemic, including its role in proving the efficacy of PrEP, and discuss the opportunities and challenges of implementing PrEP in a middle-income country.

**Discussion:**

Thailand was one of the first countries in the world to successfully reverse a generalized HIV epidemic. Despite this early success, HIV prevalence has remained high among people who inject drugs and has surged among men who have sex with men (MSM) and transgender women (TGW). Two pivotal trials that showed that the use of oral antiretroviral medication as PrEP can reduce HIV transmission were conducted partially or entirely at Thai sites. Demonstration projects of PrEP, as well as clinical trials of alternative PrEP regimens, began or will begin in 2014–2015 in Thailand and will provide additional data and experience on how to best implement PrEP for high-risk individuals in the community. Financing of drug costs, the need for routine laboratory monitoring and lack of awareness about PrEP among at-risk groups all present challenges to the wider implementation of PrEP for HIV prevention in Thailand.

**Conclusions:**

Although significant challenges to wider use remain, PrEP holds promise as a safe and highly effective method to be used as part of a combined HIV prevention strategy for MSM and TGW in Thailand.

## Introduction

Thailand, located in Southeast Asia with a population of 65.5 million [1], has a unique position in the global response to the HIV epidemic. It has the highest adult HIV prevalence (1.2% in 2012) in Asia [2], while at the same time being hailed as the first nation to successfully control and reverse a generalized HIV epidemic [3].

In the early 1990s, Thailand faced a generalized HIV epidemic, with national HIV prevalence peaking at 4.0% among male army recruits in 1993 [4] and 2.6% among pregnant women attending antenatal clinics in 1995 [5]. Much credit for reversing the epidemic has been given to the National HIV/AIDS Control Program, formed in 1991, with strong national leadership, and centred on the “100% condom use during commercial sex” program [6–10]. Since 2005, national sentinel surveillance has shown HIV prevalence of 0.5% among male military recruits and 0.5–0.6% among pregnant women [11].

**Figure 1:**
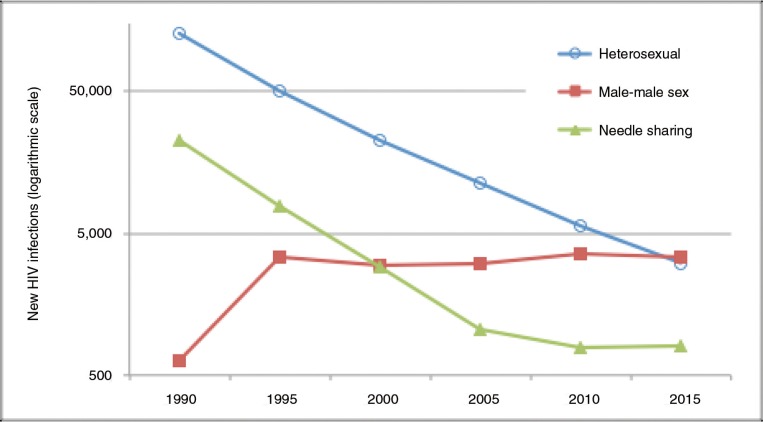
Annual new HIV infections in Thailand by risk category, 1988–2015. Adapted from Ref. [12].

While Thailand has had great success in addressing HIV transmission among the heterosexual population, emerging epidemics of HIV infection were recognized among men who have sex with men (MSM) and among transgender women (TGW) in the past decade. Although the number of new HIV infections among people who inject drugs (PWID) and heterosexuals has declined dramatically, the number of new infections among MSM has slowly increased (Figure 1). In 2003, a cross-sectional venue-based survey in Bangkok found an HIV prevalence of 17.3% among MSM [13] and in 2005 an HIV prevalence of 13.5% was found among TGW enrolled from entertainment and other venues in Bangkok, Chiang Mai and Phuket [14, 15]. By 2007, the HIV prevalence among MSM in Bangkok had almost doubled to 30.8% [16]. Average HIV prevalence among TGW in the three cities of Bangkok, Chiang Mai and Phuket in 2010 was 10.1% [17]. HIV incidence rates, especially among young MSM aged 18–21 years, are alarmingly high: 8.4 per 100 person-years (PY) in Chiang Mai (2008–2009) [18], 9.7 per 100 PY in Pattaya (2009–2010) [19] and 12.2 per 100 PY in Bangkok (2005–2011) [20].

From a generalized epidemic driven largely by heterosexual sex and the male clients of female sex workers in the 1990s, by 2015 the HIV epidemic in Thailand had evolved into a concentrated epidemic in which the MSM population is the most affected [11].

Pre-exposure prophylaxis (PrEP) with antiretroviral drugs has been proven to be effective in reducing the transmission of HIV infection among MSM and other key populations at risk. In this paper, we describe the unique role that Thailand has played in the global effort to combat the HIV epidemic, including its role in proving the efficacy of PrEP, and discuss the opportunities and challenges of implementing PrEP in the country.

## Discussion

### HIV prevention research in Thailand

Having participated in seven HIV preventive Phase III efficacy trials, Thailand ranks second only to South Africa worldwide in the number of biomedical HIV prevention trials conducted within the country [3]. It is also the only country in the world to host Phase III trials showing efficacy of a prime-boost HIV vaccine in the general population [21] and of oral PrEP in two distinct high-risk populations, MSM and PWID [22, 23].

Completed and ongoing PrEP clinical trials and demonstration projects in Thailand are listed in Table 1. The iPrEx study was the first clinical trial to show efficacy (44% reduction in HIV infection over all sites) of oral PrEP using a combination of tenofovir disoproxil fumarate (TDF) and emtricitabine (FTC) [22]. The study enrolled 2499 MSM and TG participants in six countries, of whom 114 enrolled in the Prevention of Infection in Man (PIMAN) Clinic in Chiang Mai, Thailand. The efficacy of PrEP was highly dependent on adherence; fewer than half of the subjects in the active treatment arm of the study had detectable serum levels of study drugs, but those that did had a 92% reduction in HIV infection risk.

**Table 1:** PrEP clinical trials and demonstration projects implemented and planned in Thailand

Project title	Time period	Project design	Locations	Thai agency	Intervention	N Thailand (total)	Ref.
iPrEX	2007–2009	Phase III Clinical trial	Chiang Mai	PIMAN, Chiang Mai University	TDF-FTC daily	114 (2499) MSM, TGW	[22]
BTS	2005–2012	Phase III Clinical trial	Bangkok	TUC, BMA	TDF daily	2413 (2413) PWID	[23]
HPTN 067	2011–2014	Phase II Clinical trial	Bangkok	TUC	TDF-FTC daily vs. intermittent	193 (622) MSM, TGW	[25]
MTN 017	2013–	Phase II Crossover trial	Bangkok, Chiang Mai	TUC, Chiang Mai University	TDF-FTC daily vs. daily/ intermittent rectal gel	54 (186) MSM, TGW	[26]
PrEP-30	2014–	Demonstration	Bangkok	Thai Red Cross AIDS Research Center	Self-paid TDF-FTC daily	Unlimited	N/A
Test, treat and prevent	2015–	Implementation science	Bangkok+4 cities	Thai Red Cross AIDS Research Center	TDF-FTC daily	2000 (2000) MSM, TGW	[36]

PIMAN, Prevention of Infection in Man; TUC, Thailand Ministry of Public Health – US Centers for Disease Control and Prevention Collaboration; BMA, Bangkok Metropolitan Administration.

The Bangkok Tenofovir Study (BTS) evaluated oral PrEP with tenofovir alone in 2413 PWID at 17 drug treatment clinics in Bangkok and showed a 49% reduction in HIV incidence [23]. Again, adherence to the study medicine was important; efficacy increased to 56% among participants who reported taking study medication ≥71% of the time, and to 74% among those with detectable plasma tenofovir.

Thai sites continue to participate in ongoing PrEP clinical trials. The HIV Prevention Trials Network (HPTN) 067 study is a Phase II, three-armed randomized trial of the pharmacokinetics and behavioural aspects of intermittent versus daily oral TDF-FTC for the prevention of HIV infection among MSM, TGW and high-risk heterosexual women [24]. The study enrolled 193 MSM and TGW in Thailand, 238 in the United States, and 191 high-risk heterosexual women in South Africa. However, the data reported thus far do not disaggregate MSM and TGW and it remains to be seen if the trial included a significant number of TGW participants. Two Thai sites, in Bangkok and Chiang Mai, are included in the Microbicide Trials Network (MTN) 017 study, a Phase II randomized crossover trial of the safety and acceptability of daily or sexual event–driven 1% tenofovir reduced glycerin rectal gel versus daily oral TDF-FTC [25].

A notable gap in the evidence base for oral PrEP is the very low number of TGW who have participated in PrEP trials to date [26]. Transgender individuals in Thailand and elsewhere, both male-to-female and female-to-male, experience exposure to genital reassignment surgery and hormone use with as yet unknown effects on HIV risk or on the efficacy or oral PrEP [26, 27]. More research is needed on the role of PrEP for HIV prevention in this unique population before it can be concluded that PrEP will be as efficacious as it is in other high-risk populations.

### Acceptability of PrEP among high-risk populations in Thailand

Knowledge about PrEP among Thai MSM has been reported to vary greatly, from 7% in an online survey [28] to 66% of MSM recruited in entertainment venues in a large urban area [29]. The latter survey was conducted in Chiang Mai City, which hosted one of the sites that implemented the iPrEx study and may therefore account for the higher awareness of PrEP in that location.

Three surveys have found that willingness to use PrEP was 36–41% among Thai MSM and TGW [28–30]. One survey reported that 85% of MSM said that they would “probably” or “definitely” use PrEP if it were available [30]. One-quarter (24%) of online respondents said that they would not take PrEP even if it were offered for free, but 65% stated that they would be willing to pay for PrEP medication [28]. Most (65%) of those were willing to pay a maximum of only 750 Thai Baht (THB), or about US$25, per month for PrEP. When given a choice between routes of PrEP administration, the majority chose a daily oral pill over event-driven oral administration or intermittent intramuscular injections [30].

Rectal microbicides have not yet been proven to decrease the risk of HIV transmission, but are in development, and Phase II clinical trials are being conducted in Thailand and other countries [25]. Among a cohort of MSM in Bangkok, 79% said that they would be willing to participate in an efficacy trial of a rectal microbicide and 97% would be willing to use a rectal gel if it were found to be effective [31].

There have been no published reports of willingness to use PrEP among PWID in Thailand. In addition, neither the World Health Organization nor the Thai advocacy groups such as the Thai AIDS Treatment Action Group have thus far publicly endorsed PrEP for PWID [32, 33].

### PrEP implementation projects in Thailand

Both the iPrEx trial and the BTS included open-label extensions of PrEP for study participants after the efficacy trials were completed, which provide initial experience in implementing PrEP in Thailand. In each trial, HIV-negative participants were informed of the study results and offered drug and follow-up free of charge for one to two years.

The iPrEx trial enrolled 114 participants in Chiang Mai, Thailand. Of these, 61 (54%) HIV-negative MSM continued in the extension phase after completion of the main study and 54 (89%) of these chose to take oral TDF-FTC as PrEP for up to an additional 18 months [34]. Follow-up visits took place every 4–12 weeks at a community-based clinic. Adherence in the extension phase was high: 43/54 (80%) of Thai MSM tested had detectable plasma tenofovir, higher than the combined 71% of subjects worldwide. Study staff noted that participants perceived a clear benefit to PrEP use and were more open to discussing and problem-solving difficulties with adherence than they were during the clinical trial phase of the study, when they did not know if they were receiving drug or placebo and adherence was monitored more stringently (S. Chariyalertsak, personal communication, 2014).

The BTS extension provided open-label PrEP for one year via directly observed treatment (DOT) to study participants in the 17 drug treatment clinics and in prisons. A total of 1327 BTS participants returned to receive study results and 785 (59%) chose to take TDF [35]. Among the 128 incarcerated participants, 94% missed fewer than eight doses of TDF during the previous 28 days at their most recent assessment. Among non-incarcerated subjects, however, only 15% met this adherence threshold. Daily PrEP was dispensed as DOT in drug treatment clinics and the need for daily travel may be responsible, in part, for the low adherence among BTS participants in the community. The study investigators concluded that additional adherence support and distribution mechanisms may be needed to make PrEP more acceptable and accessible to non-incarcerated PWID.

Two additional PrEP demonstration projects are scheduled to begin providing PrEP to MSM and TGW in Bangkok and several other cities in early 2015. The Test, Treat, and Prevent HIV program is supported by the Thailand Ministry of Public Health and PEPFAR and plans to enrol 8000 high-risk (defined as having anal intercourse without using a condom at least once in the previous six months) MSM and TGW, including sex workers [36, 37]. Participants testing HIV positive will be offered antiretroviral therapy (ART) regardless of CD4 count and 600 of those testing negative will be offered PrEP with one-year follow-up. The trial will be conducted at both facility- and community-based sites.

The Thai Red Cross AIDS Research Centre in Bangkok, which operates the largest HIV testing and counselling centre in Thailand, also plans to implement a demonstration project of fee-based PrEP for all high-risk individuals, including MSM, TGW, heterosexuals and PWID. The PrEP-30 project aims to evaluate the feasibility of self-pay PrEP as a sustainable model not reliant on government or external funding. A complete HIV prevention package, including locally produced TDF-FTC, counselling and laboratory testing will be provided for a fee of 30 THB per day, or about US$30 per month. Although Thailand is an upper middle-income country with a per capita income of US$445 per month [38], the willingness of at-risk individuals to pay out of pocket for PrEP remains untested.

The Thai government along with national and international non-governmental organizations held a meeting with Thai experts and stakeholders in August 2012 to discuss the use of ARV drugs for HIV prevention [39]. One conclusion of the meeting was that the goal of an AIDS-free Thailand could not be achieved through the expansion of the existing behavioural interventions alone, and that the use of ARV drugs through expanded treatment and PrEP would eventually become part of the national AIDS strategy. Concerns raised in regard to PrEP included stigma and discrimination reducing access to key populations, the cost of drugs, lack of spare capacity in the healthcare system, the risk of side effects when giving drugs to a healthy population and the potential for HIV resistance to develop. Among the recommendations that were made to the Ministry of Public Health were to implement operational research on the use of PrEP for key affected populations and to consider task-shifting for service delivery.

### Opportunities and challenges for PrEP implementation in Thailand

The HIV epidemic among MSM in Thailand continues unabated with high incidence and prevalence. Similar to experiences elsewhere, past and current HIV prevention programming has had limited efficiency or success in decreasing the transmission of HIV in this population [40]. The Test and Treat strategy has the potential to decrease HIV transmission in the community by the early recognition and treatment of HIV infection, but the numbers (or proportion) of MSM covered by Test and Treat programs at present will not be enough to affect the overall epidemic. New and expanded HIV prevention modalities are needed if the HIV epidemic among MSM is to be controlled. The Thai National Guidelines on HIV/AIDS Treatment and Prevention were revised in 2014 and for the first time recommended PrEP for key populations [41].

Significant challenges to the wider use of PrEP remain, the foremost being finding the resources, both human and financial, that would be required to make PrEP available to all potential users in Thailand. Even with the availability of low-cost generic TDF-FTC, drug costs will be substantial if tens of thousands of otherwise healthy MSM start taking daily PrEP for an indefinite period of time. Thailand recently revised its National Guidelines on HIV/AIDS Treatment and Prevention to recommend treatment for all people living with HIV (PLHIV), regardless of CD4 count. This change alone will increase the number of Thai PLHIV eligible for ART from 246,000 to 407,000 in 2015, an increase of over 65% [11]. The use of limited resources to provide ART to all currently eligible PLHIV may preclude significant expansion of publicly-funded antiretroviral-based HIV prevention strategies at any time in the near future.

Health care personnel and facilities need to prepare for providing PrEP to an at-risk population that could number in the tens or hundreds of thousands. Training needs for health care workers include knowledge about PrEP, risk-reduction counselling skills and importantly the promotion of non-discriminatory attitudes towards PrEP and towards individuals who engage in stigmatized, high-risk behaviour. Experience from PrEP clinical trials and demonstration projects in Thailand can provide examples of best practices to help guide expanded PrEP programming. ART clinics, many of which are already overburdened with high caseloads of PLHIV, may not be the optimal places to provide PrEP services. HIV testing and community-based centres may offer alternate locations to provide PrEP to at-risk populations.

Additional challenges to PrEP implementation include limited knowledge and lack of awareness about PrEP in at-risk communities, ensuring adherence, side effects of the medications, the need for laboratory monitoring and frequent HIV testing, and the risk for the emergence of HIV drug resistance. Demonstration projects using implementation science methodology will be useful to ascertain the most appropriate approaches to the expansion of PrEP services in Thailand and other developing countries [42].

### Recommendations for research agenda

With only limited experience in the implementation of PrEP in Thailand, numerous questions remain, including the appropriate role for PrEP within the national HIV strategy and the most practical and cost-effective ways to provide PrEP services. Which individuals within at-risk groups will benefit the most from the use of PrEP? Is PrEP as efficacious for TGW as it is for other at-risk groups? How to best support adherence, especially among PWID? Who will cover the cost of PrEP drugs in middle-income countries? Will PrEP users be willing to pay for it? What is the role of community-based services and task-shifting to non-physician providers?

These questions and others will need to be addressed as new and ongoing projects in Thailand provide practical experience implementing PrEP. Lessons learned can also be applied to other countries in Asia, which have far less experience with PrEP implementation but have similar challenges in scaling up biomedical HIV prevention methods and face similar HIV epidemics among PWID, MSM and TGW [43].

## Conclusions

Thailand needs new and innovative HIV prevention strategies to address the rapidly evolving HIV epidemic among MSM and TGW. Oral PrEP has been proven to be effective at reducing HIV transmission among MSM, as well as among heterosexuals and PWID. Evidence on the efficacy of PrEP for TGW is lacking and Thailand, with a large population of TGW and a number of facilities that provide sexual reassignment surgery, is well placed to lead the agenda in determining the proper role of PrEP, if any, in this population.

Demonstration projects planned for implementation in 2015 will provide further evidence on the feasibility, acceptability and financial viability of PrEP provision for high-risk MSM and TGW in Thailand. However, PrEP is just one part of a combination HIV prevention strategy. HIV testing and counselling will remain a key entry point to HIV prevention programming, to HIV treatment and care services for those who test HIV positive and to PrEP for those identified as at-risk and eligible. Increased efforts are needed to encourage and attract higher numbers of MSM and TGW to access HIV testing in Thailand.

The BTS demonstrated PrEP efficacy for PWID. Furthermore, the extension phase of the study showed that DOT PrEP can be successfully continued in correctional settings [35]. To demonstrate the continued delivery of this intervention to those who became incarcerated during follow-up is an important outcome of the BTS study with potential implications for addressing the high rate of HIV transmission that has been documented in prison populations [44–47].

Although PrEP has proven efficacy in the clinical trial setting in Thailand and elsewhere, challenges to implementation and questions about the optimal use of TDF-FTC for PrEP remain. It has yet to be scaled up to or evaluated at the level that would be necessary to slow down or halt the epidemic of HIV among MSM in either the developed or developing world. Nonetheless, oral PrEP holds promise as an important component of a combined HIV prevention strategy.

## References

[1] National Statistics Office (2011). The 2010 population and housing census.

[2] Joint United Nations Programme on HIV/AIDS (UNAIDS) (2013). HIV in Asia and the Pacific.

[3] van Griensven F, Phanuphak N, Srithanaviboonchai K (2014). Biomedical HIV prevention research and epidemic control in Thailand: two sides of the same coin. Sex Health.

[4] Royal Thai Army (2013). Results of biannual HIV surveillance among Thai military recruits, 1989–2012.

[5] Ministry of Public Health (2012). Results of sentinel HIV surveillance in Thailand. Round 1–30, 1988–2012.

[6] Rojanapithayakorn W (2006). The 100% condom use programme in Asia. Reprod Health Matters.

[7] Rojanapithayakorn W, Hanenberg R (1996). The 100% condom program in Thailand. AIDS.

[8] The World Bank (2000). Thailand's response to AIDS: building on success, confronting the future.

[9] Punpanich W, Ungchusak K, Detels R (2004). Thailand's response to the HIV epidemic. Yesterday, today and tomorrow. AIDS Educ Prev.

[10] World Health Organization (WHO) (2004). Experiences of the 100% condom use program in selected countries in Asia.

[11] Thai National AIDS Committee (2014). Thailand ending AIDS: 2014 Thailand AIDS response progress report.

[12] Thai Working Group for HIV/AIDS Projections (2010). Projection for HIV/AIDS in Thailand 2010–2030.

[13] van Griensven F, Thanprasertsuk W, Jommaroeng R, Mansergh G, Naorat S, Jenkins RA (2005). Evidence of a previously undocumented epidemic of HIV infection among men who have sex with men in Bangkok, Thailand. AIDS.

[14] van Griensven F, Varangrat A, Wimolsate W, Tappero JW, Sinthuwattanawibol C, McNicholl JM (2006). HIV prevalence among populations of men who have sex with men-Thailand, 2003 and 2005. Morb Mortal Wkly Rep.

[15] Guadamuz TE, Wimonsate W, Varangrat A, Phanuphak P, Jommaroeng R, McNicholl JM (2011). HIV prevalence, risk behavior, hormone use, surgical history and HIV infection among transgender persons in Thailand. AIDS Behav.

[16] van Griensven F, Varangrat A, Wimonsate W, Tanpradech S, Kladsawad K, Chemnasiri T (2010). Trends in HIV prevalence, estimated HIV incidence and risk behavior among men who have sex with men in Bangkok, Thailand, 2003–2007. J Acquir Immune Defic Syndr.

[17] Bureau of Epidemiology (2012). Results of venue-based HIV surveillance among men who have sex with men in Thailand, 2012.

[18] Chariyalertsak S, Kosachunhanan N, Saokhieo P, Songsupa R, Wongthanee A, Chariyalertsak C (2011). HIV incidence, risk factors, and motivation for biomedical intervention among gay, bisexual men, and transgender persons in Northern Thailand. PLoS One.

[19] Nitayaphan S, Benenson M, Sriplienchan S, Morgan P, Eamsila C, Chiu J (2010). ECHO Study (Early Capture HIV Cohort): efficient detection of acute HIV-1 infections in Pattaya, Thailand (RV 217).

[20] Ananworanich J, Chitwarakorn A, Wimonsate W, Varangrat A, Chaikummao S, Sriporn A (2013). HIV and syphilis infection among men who have sex with men – Bangkok, 2005–2011. Morb Mortal Wkly Rep.

[21] Rerks-Ngarm S, Pitisuttithum P, Nitayaphan S, Kaewkungwal J, Chiu J, Paris R (2009). Vaccination with ALVAC and AIDSVAX to prevent HIV-1 infection in Thailand. N Engl J Med.

[22] Grant RM, Lama JR, Anderson PL, McMahan V, Liu AY, Vargas L (2010). Preexposure chemoprophylaxis for HIV prevention in men who have sex with men. N Engl J Med.

[23] Choopanya K, Martin M, Suntharasamai P, Sangkum U, Mock PA, Leethochawalit M (2013). Antiretroviral prophylaxis for HIV infection in injecting drug users in Bangkok, Thailand (the Bangkok Tenofovir Study): a randomised, double-blind, placebo controlled phase 3 trial. Lancet.

[24] HIV Prevention Trials Network (HPTN) (2012). HPTN 067. The ADAPT study.

[25] Microbicide Trials Network (MTN) (2013). MTN-017.

[26] van Griensven F, Ayutthaya P, Wilson E (2013). HIV surveillance and prevention in transgender women. Lancet Infect Dis.

[27] Gooren LJ, Sungkaew T, Giltay EJ, Guadamuz TE (2015). Cross-sex hormone use, functional health and mental well-being among transgender men (Toms) and Transgender Women (Kathoeys) in Thailand. Cult Health Sex.

[28] Sineath RC, Finneran C, Sullivan P, Sanchez T, Smith DK, Griensven F (2013). Knowledge of and interest in using preexposure prophylaxis for HIV prevention among men who have sex with men in Thailand. J Int Assoc Provid AIDS Care.

[29] Yang D, Chariyalertsak C, Wongthanee A, Kawichai S, Yotruean K, Saokhieo P (2013). Acceptability of pre-exposure prophylaxis among men who have sex with men and transgender women in Northern Thailand. PLoS One.

[30] Wheelock A, Eisingerich AB, Ananworanich J, Gomez GB, Hallett TB, Dybul MR (2013). Are Thai MSM willing to take PrEP for HIV prevention? An analysis of attitudes, preferences and acceptance. PLoS One.

[31] Thienkrua W, Todd CS, Chaikummao S, Sukwicha W, Yafant S, Tippanont N (2014). Prevalence and correlates of willingness to participate in a rectal microbicide trial among men who have sex with men in Bangkok. AIDS Care.

[32] Treatment Action Group U.S. Centers for Disease Control and Prevention (CDC) Sponsored HIV Preexposure Prophylaxis (PrEP) Trial among Thai Injection Drug Users Marred by Lack of Response to Community Concerns: Statement of Thai Drug Users Network (TDN), Thai AIDS Treatment Action Group (TTAG), and Treatment Action Group (TAG) [Internet].

[33] WHO (2014). Consolidated guidelines on HIV prevention, diagnosis, treatment, and care for key populations.

[34] Grant RM, Anderson PL, McMahan V, Liu A, Amico KR, Mehrotra M (2014). Uptake of pre-exposure prophylaxis, sexual practices, and HIV incidence in men and transgender women who have sex with men: a cohort study. Lancet Infect Dis.

[35] Martin M, Vanichseni S, Suntharasamai P, Sangkum U, Mock PA, Leethochawalit M Enrollment and preliminary follow-up of injecting drug users receiving pre-exposure prophylaxis in Bangkok.

[36] Study to evaluate the feasibility of community-based test and treat strategies among men who have sex with men and transgender women to increase the uptake of HIV testing and treatment services in Thailand [Internet].

[37] Evaluation of a facility-based test, treat, and prevent HIV program among men who have sex with men and transgender women in Thailand [Internet]. http://www.clinicaltrials.gov/ct2/show/NCT02369887.

[38] The World Bank Thailand [Internet].

[39] National consultation on the strategic use of ARVs – Thailand. Meeting report (2012).

[40] Beyrer C, Baral SD, van Griensven F, Goodreau SM, Chariyalertsak S, Wirtz AL (2012). Global epidemiology of HIV infection in men who have sex with men. Lancet.

[41] Essentials of HIV/AIDS treatment and prevention 2014 [cited 2015 March 25] Thailand [Internet].

[42] World Health Organization (2012). Guidance on oral pre-exposure prophylaxis (PrEP) for serodiscordant couples, men and transgender women who have sex with men at high risk of HIV: recommendations for use in the context of demonstration projects.

[43] Lo YR, Kato M, Phanuphak N, Fujita M, Duc DB, Sopheap S (2014). Challenges and potential barriers to the uptake of antiretroviral-based prevention in Asia and the Pacific region. Sex Health.

[44] Jurgens R, Nowak M, Day M (2011). HIV and incarceration: prisons and detention. J Int AIDS Soc.

[45] Vanichseni S, Kitayaporn D, Mastro TD, Mock PA, Raktham S, Des Jarlais DC (2001). Continued high HIV-1 incidence in a vaccine trial preparatory cohort of injection drug users in Bangkok, Thailand. AIDS.

[46] Thaisri H, Lerwitworapong J, Vongsheree S, Sawanpanyalert P, Chadbanchachai C, Rojanawiwat A (2003). HIV infection and risk factors among Bangkok prisoners, Thailand: a prospective cohort study. BMC Infect Dis.

[47] Buavirat A, Page-Shafer K, van Griensven GJP, Mandel JS, Evans J, Chuaratanaphong J (2003). Risk of prevalent HIV infection associated with incarceration among injecting drug users in Bangkok, Thailand: case-control study. Br Med J.

